# A Comparative Study between Karydakis Flap Reconstruction and Double Z-Plasty in Patients with Sacrococcygeal Pilonidal Disease

**DOI:** 10.1155/2014/523015

**Published:** 2014-09-07

**Authors:** Ankit Kayal, Akhlak Hussain, Anil Choudhary, Ashok Meghwal

**Affiliations:** Department of General Surgery, JLN Medical College and Hospital, Ajmer, Rajasthan 305001, India

## Abstract

The most difficult problems that follow surgery for pilonidal disease are persistent unhealed midline wound and recurrence. Various innovations were proposed to deal with these problems. The adipocutaneous flap of Karydakis was devised to shift the natal cleft, while Z-plasty involves fasciocutaneous flap. The present prospective randomized trial was conducted on 50 cases of symptomatic or recurrent pilonidal sinuses divided randomly into two equal groups undergoing Karydakis procedure and Z-plasty. The duration of hospitalization for Karydakis procedure was found significantly lesser than that for double Z-plasty (*P* < 0.001). Similar observations are obtained for duration of wound healing (*P* < 0.001), work off period (*P* < 0.001), and the duration of presence of significant pain postoperatively (*P* < 0.001). The overall complications were more in double Z-plasty. Recurrence developed in 32% of the cases in double Z-plasty group comparable to no recurrence seen in Karydakis procedure. Thus, Karydakis flap was found superior to double Z-plasty having less seroma formation, no local hematoma, and no flap necrosis. Statistically, this comparison was highly significant (*P* < 0.001). Karydakis flap has some added advantages over double Z-plasty technique like keeping scar away from the midline and flattening of the natal cleft, thus reducing local recurrence rates.

## 1. Introduction

Strong evidence shows that pilonidal disease originates in the epidermis, in a midline stretched hair follicle [1]. Early pilonidal changes are amplified due to deep tissue disruption from moisture, anaerobic conditions, hairs, and bacteria. On the basis of these observations, a new paradigm of origin emerges that pilonidal disease is exclusively an epidermal problem, rather than a deep tissue problem, which is the theory behind current recommendations for wide excision [2, 3]. Therefore, the focus of treatment should be actions to change the conditions that attack epidermis, rather than wide excision which attacks deep and healable tissue.

The most difficult complication that may follow surgery for pilonidal disease is a persistent unhealed midline wound, most commonly seen after laying open or excision of primary disease. Although the authors are familiar with all procedures, Karydakis and Z-plasty procedures are among the common procedures done currently, so they were chosen in the study. Karydakis procedure is basically a gluteal advancement flap [4]. Unlike other flap procedures (i.e., rhomboid flap, Z-plasty, and gluteal rotation flap) which are fasciocutaneous, Karydakis flap is adipocutaneous flap; it is, therefore, technically easier, less bloody, and less time consuming; it obviously has a better cosmetic outcome as it leaves a single, lateral, longitudinal scar; and it requires significantly shorter hospital stay. In addition, the natal cleft may satisfactorily be flattened by Karydakis procedure. The present study was done to study Karydakis flap reconstruction as a treatment of symptomatic pilonidal sinus disease and to compare Karydakis flap reconstruction and double Z-plasty as a treatment of symptomatic pilonidal sinus disease.

## 2. Methods

**Figure 1:**
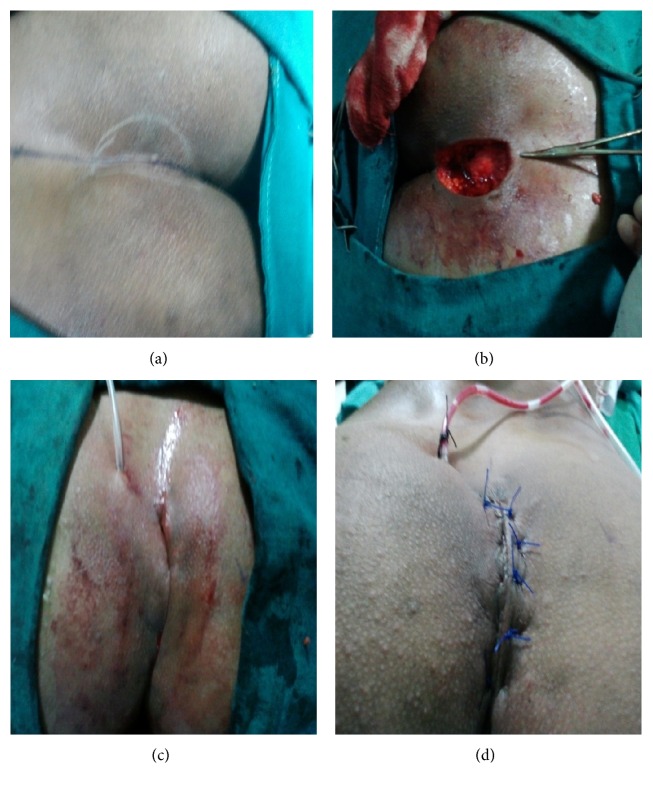
Karydakis procedure: (a) incision marking, (b) after excision, (c) initial approximation, and (d) complete procedure.

**Figure 2:**
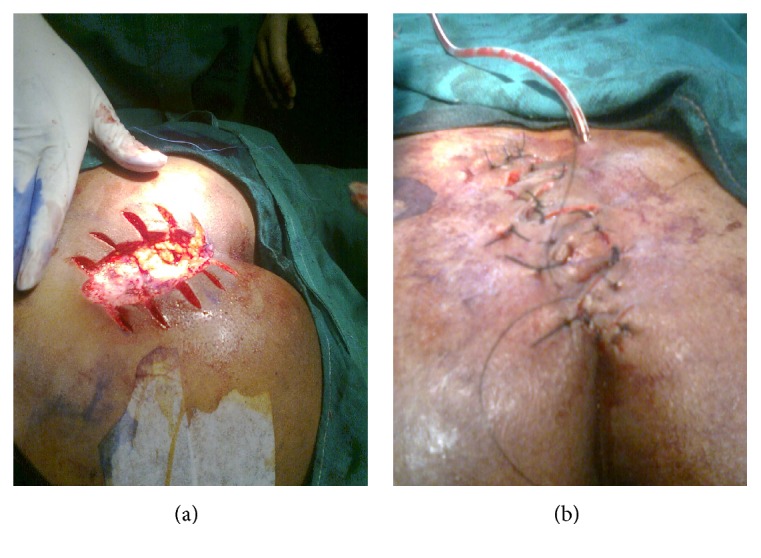
Z-plasty (a) postexcision picture and (b) complete procedure.

With proper ethical permission, a prospective randomized trial was conducted on 50 cases of symptomatic or recurrent pilonidal sinuses reported in outpatient department of J.L.N. Medical College and Hospital, Ajmer, from July 2009 till December 2011. Patients are recruited consecutively and divided randomly into two groups of 25 patients each. The sample size is decided according to the past experience of cases of pilonidal sinuses and local guidelines. The cases are randomized on the basis of hospital registration number by the initial screening personnel who did not get involved in operative part. After proper preoperative workup and antibiotic prophylaxis, first group (Gp 1) undergoes wide excision with Karydakis flap reconstruction and the second group undergoes wide excision with double Z-plasty. Authors were having good experience in both cases. Karydakis technique consisted of a vertical eccentric elliptical incision extended down to the postsacral fascia; complete removal of unhealthy tissue, including a rim of normal tissue around the cyst and sinus tract; mobilization of the medial wound edge by undercutting the adipose tissue at a depth of 1 cm; advancement of flap across the midline to the postsacral fascia; and suturing of its edges to the lateral wound edges. Close attention was paid to ensure adequate mobilization and tension free fixation of flap, which was lateralized ≥1 cm from midline (Figures 1(a)–1(d)). In group 2 (Gp 2), Z-plasty was performed by a vertical elliptical incision extended down to the postsacral fascia; complete removal of unhealthy tissue with a rim of normal healthy tissue; two oblique incisions given by making an angle of 60 degrees with the vertical line (Figures 2(a) and 2(b)). The flaps are suitably interdigitated and sutured in three layers according to depth of wound so as to avoid negative spacing. All patients were asked to pay attention to hygiene rules, to not sit or use a semisitting position for 15 days, and to depilate hairs around their gluteal region as necessary for every 3 months beginning after hospital discharge.

Data obtained during hospital admission and at 10, 21, and 30 days after discharge included complications, duration of hospitalization healing, work off period, and degree of satisfaction. Persistence of pain was asked for during every visit as significant or nonsignificant. Significant pain was taken as that which interferes significantly with activities of daily living. The surgical procedure was considered “unsuccessful” (early failure) if a given patient experienced purulent discharge, abscess, or complete wound dehiscence for which further treatment was required within 30 days of the operation. All similar events that occurred following an uneventful recovery period were considered “recurrent disease.” The data was analysed using unpaired Student's *t*-test and the proportions were analysed using Fisher exact test.

## 3. Results

The most common age of presentation in both groups was 21–30 years (mean age: 28 years). In both groups, the incidence in male patients was 9 times more than that in females. In both groups, the incidence was more common in patients with coarse body hair density (80–84%) and among the patients with a deep natal cleft (88%). Grossly, primary disease was present in 82% of the patients among which 87.8% were males. Recurrent disease was present in 20% of patients and all of them are males and all these patients have deep natal cleft. There were 4 recurrent cases included in group 1 and 5 in group 2. Due to randomization, this factor did not significantly affect the study results. In both groups, the common clinical presentations were small pus discharging sinus (56%), multiple discharging sinus (28%), and swelling with pus discharging sinus (16%).

The duration of hospitalization for Karydakis procedure was significantly lesser than that for double Z-plasty (mean: 4 versus 11 days, resp., *P* < 0.001). Similar observations are obtained for duration of wound healing (mean: 13 versus 30 days, resp., *P* < 0.001), work off period (mean: 11 versus 36 days, resp., *P* < 0.001), and the duration of presence of significant pain postoperatively (mean: 11 versus 38 days, resp., *P* < 0.001). The overall complications were more in double Z-plasty. The complications observed in double Z-plasty were infection of wound (60%), seroma (56%), hematoma (52%), necrosis (48%), wound dehiscence (48%), and loss of sensation (40%). The complications in Karydakis procedure were infection (16%), seroma (12%), and loss of sensation (4%). There was absence of hematoma, necrosis, and wound dehiscence in Karydakis procedure. Recurrence developed in 32% of the cases in double Z-plasty group comparable to no recurrence seen in Karydakis procedure, but the patients were followed up until 6 months. Recurrence information was based on the voluntary information by the patients after that in the outpatient department. 89% of complications occur in male patients and the remaining 11% in females. All these complications were more common among patients with primary disease, deep natal cleft, and coarse body hair density (Table 1).

## 4. Discussion

Pilonidal sinus disease is an acquired condition usually seen in young adults and carries high postoperative morbidity and patient's discomfort. Through this study, we compare the two modern methods of treatment, namely, Karydakis procedure and double Z-plasty.

(1) Epidemiology: in present study, pilonidal sinus disease is an acquired condition usually seen in young adults of 21 to 30 years of age. The same has been observed by Z.S. Matar (15 to 32 years) and M.R.B. Keighley (peak age incidence is 15 to 24 years) [5, 6]. This higher occurrence in the 3rd decade of life is because pilonidal disease starts at the onset of puberty, when sex hormones start acting on pilosebaceous glands in the natal cleft. A hair follicle becomes distended with keratin and subsequently infected, leading to a folliculitis and an abscess which extends into the subcutaneous fat. Similar to our study, Chintapatla et al. showed male preponderance of this disease [7]. This may be mainly due to their more hirsute nature. Awad and Saad reported that 56.3% of pilonidal disease patients had coarse hair density and deep natal cleft [8]. We fully agree with this, as we found that patients with recurrent disease were all males having a coarse body hair density and deeper natal cleft.
(2) Postoperative comparison: hospitalization, wound healing, and work off periods are relative measures of outcome. They are strongly related to personal, sociocultural, and socioeconomic levels, type of job, social assurance, and behavioral patterns. We observed shorter hospitalization period with Karydakis flap technique (4.08 + 0.91 days) in comparison to double Z-plasty (10.92 + 2.29 days). In the present study, the mean duration of wound healing was 12.84 + 1.70 days, the number of days in which pain lasted postoperatively was 11.52 + 1.50 days, and work off periods were 10.56 + 1.29 days in Karydakis flap procedure, while in double Z-plasty technique the mean days of wound healing were 30.16 ± 8.97 days, the number of days in which pain lasted postoperatively was 37.92 ± 8.31 days, and work off periods were 35.72 ± 8.59 days. Fazeli et al. (2006) in a clinical trial of 216 cases divided into three arms of 72 cases each have concluded that wounds healed much faster in the Z-plasty group, 15.4 days, and the work off periods were 17.5 days [9]. Taubanakis in 1986 produced a study in which he found that mean time of healing in Z-plasty was <14 days [10].
(3) Complications: in present study, the overall complication rates were maximum for the double Z-plasty group (45.07%) and the commonest complications were loss of local sensation (68%), tip necrosis (44%), infection (8%), seroma formation (4%), and wound dehiscence (4%). Similarly, Sayed, in his experience of 30 cases with Z-plasty, reported among early complications loss of local sensation in 58%, tip necrosis in 20%, wound infection in 6.7%, and wound dehiscence in 3.3% [11]. Loss of local sensation is a known complication following Z-plasty because in this technique flaps are transposed in such a way to avoid suture line in midline. To achieve this, we have to make incision, thereby cutting cutaneous nerves. Mansoory and Dickson, in their experience of 127 cases with Z-plasty, suggested that more than 50% of patients develop loss of local sensation and over 30% of patients develop tip necrosis [12]. Tip necrosis was also more with our technique of double Z-plasty (44%); it was because of sharp triangles. However, it was only superficial necrosis, thereby increasing morbidity, but it does not affect overall results. In our experience, hematoma occurred in cases having deep natal cleft and coarse body hair density. This may be attributed to more dissection in deep natal cleft cases and minimal use of cautery in order not to compromise the blood supply of the flaps, resulting in inadequate hemostasis and hematoma formation. Necrosis was a major complication among patients who underwent treatment for the disease. This is mainly because of occurrence of compromised blood supply in making flaps. In double Z-plasty, to minimize the tip necrosis, we have observed and advise to do the following:
  (i) the incision to achieve transposition of flaps should be in full depth having deeper tissues along with the skin;
  (ii) approximation of soft tissue (subcutaneous and deeper) should be meticulous by vicryl suture;
  (iii) the cautery should be minimally used.

However, in Karydakis flap technique, infection, seroma, hematoma, loss of sensation, flap necrosis, and wound dehiscence were very less common. In both groups, wound dehiscence and infection were more common in cases having deep natal cleft and coarse body hair density. This is because, as we already know the perineum to be the most contaminated area of our body with the mid natal cleft being the bacterial harbor where maximum bacterial colonization occurs, dense body hair adversely affects maintenance of a proper hygiene, thus increasing the infection rate. So, with our experience, it is obvious that the deep natal cleft and coarse body hair density not only are risk factors for the causation of the disease but also complicate the procedure done, increasing the infection, necrosis, and wound dehiscence rate.

(4) Recurrences: in present study, no recurrence proves the superiority of the Karydakis flap procedure over double Z-plasty technique for treatment of pilonidal disease.

## 5. Conclusion

On comparison, Karydakis flap was found superior to double Z-plasty having less seroma formation, no local hematoma, and no flap necrosis. Statistically, this comparison was highly significant (*P* < 0.001). Karydakis flap has some added advantages over double Z-plasty technique like keeping scar away from the midline and flattening of the natal cleft, thus reducing local recurrence rates. Hence, we recommend use of Karydakis flap technique on larger number of patients.

## Figures and Tables

**Table 1:** Complications in patients with pilonidal disease.

Procedure/complications	Karydakis procedure	Double Z-plasty	*χ* ^2^	*P*	Significance
Seroma	3 (12%)	14 (56%)	10.784	<0.01	S
Hematoma	—	13 (52%)	17.567	<0.001	HS
Necrosis	—	12 (48%)	15.790	<0.001	HS
Infection	4 (16%)	15 (60%)	10.272	<0.01	S
Wound dehiscence	—	12 (48%)	15.790	<0.001	HS
Loss of sensation	1 (4%)	10 (40%)	9.441	<0.01	S
